# Overexpression of S-Adenosylmethionine Synthetase in Recombinant *Chlamydomonas* for Enhanced Lipid Production

**DOI:** 10.4014/jmb.2212.12009

**Published:** 2023-01-16

**Authors:** Jeong Hyeon Kim, Joon Woo Ahn, Eun-Jeong Park, Jong-il Choi

**Affiliations:** 1 Department of Biotechnology and Bioengineering, Chonnam National University, Gwangju 61186, Republic of Korea; 2 Advanced Radiation Technology Institute, Korea Atomic Energy Research Institute, Jeongeup 56212, Republic of Korea; 3 Aquatic Plant Variety Center, National Institute of Fisheries Science, Mokpo 58746, Republic of Korea

**Keywords:** *Chlamydomonas reinhardtii*, adenosylmethionine synthetase, lipid accumulation, nitrogen starvation, salt stress

## Abstract

Microalgae are attracting much attention as promising, eco-friendly producers of bioenergy due to their fast growth, absorption of carbon dioxide from the atmosphere, and production capacity in wastewater and salt water. However, microalgae can only accumulate large quantities of lipid in abiotic stress, which reduces productivity by decreasing cell growth. In this study, the strategy was investigated to increase cell viability and lipid production by overexpressing S-adenosylmethionine (SAM) synthetase (SAMS) in the microalga *Chlamydomonas reinhardtii*. SAM is a substance that plays an important role in various intracellular biochemical reactions, such as cell proliferation and stress response, and the overexpression of SAMS could allow cells to withstand the abiotic stress and increase productivity. Compared to wild-type *C. reinhardtii*, recombinant cells overexpressing SAMS grew 1.56-fold faster and produced 1.51-fold more lipids in a nitrogen-depleted medium. Furthermore, under saline-stress conditions, the survival rate and lipid accumulation were 1.56 and 2.04 times higher in the SAMS-overexpressing strain, respectively. These results suggest that the overexpression of SAMS in recombinant *C. reinhardtii* has high potential in the industrial-scale production of biofuels and various other high-value-added materials.

## Introduction

Microalgae are autotrophic organisms that carry photosynthetic pigments and synthesize various organic substances by capturing carbon dioxide from the atmosphere. They are used in various industrial processes, such as the production of biofuels, cosmetics, and pharmaceutical raw materials, among which, biofuels have recently been receiving the most attention. When microalgae are used for biofuel production, the production yield per unit of culture area is much higher than that of grain-based and wood-based biomass and requires less water because microalgae are grown in a liquid medium. Moreover, less cultivated land is required as microalgae can be cultured in saltwater (including seawater). Furthermore, since agricultural chemicals are not necessary, the leftover biomass from the biofuel production process can also be used as feed or fertilizer. Therefore, the process is consistent with the 2050 Carbon Neutral Strategy recently announced by the Republic of Korea’s Ministry of Environment [1, 2]

Biodiesel is one of the most popular biofuels that can be produced from the lipid accumulated in microalgae. Although microalgae do not accumulate much lipid under normal living conditions, they do accumulate it when exposed to abiotic stress. Two steps are needed for microalgae to produce biofuel: growth under appropriate conditions followed by abiotic stress to accumulate lipids. Since this process is associated with problems due to high cost, a long cultivation period, and low productivity, it is essential to develop microalgae with high lipid content and stress resistance for industrial applications [2-6]. *Chlamydomonas reinhardtii*, a single-celled alga belonging to the Generally Recognized as Safe category, is used as a model organism in various fields such as algal physiology, photosynthesis, and metabolism, as well as biofuel production. Moreover, it has the advantages of easy laboratory-scale experimentation, fast cultivation, and scalability [7-9]. *C. reinhardtii* also accumulates large quantities of lipids under abiotic stress, especially in nitrogen-deficient conditions [10]. Therefore, many studies are being conducted on the industrial development of the strain. In the studies that have been conducted so far, CRISPR-Cas9 technology has been used to facilitate genetic manipulation; the ferredoxin gene *petF* was overexpressed to increase heat and H_2_O_2_ stress resistance, and putative *ACSMD* (2-amino-3-carboxymuconate-6-semialdehyde decarboxylase) from the macroalga *Pyropia yezoensis* was transferred to *C. reinhardtii* to increase stress resistance in nitrogen-deficient conditions and enhance lipid production [11-13].

S-Adenosylmethionine (SAM) is a cofactor in most organisms and is synthesized from adenosine triphosphate (ATP) and methionine by the action of SAM synthase (SAMS). SAM acts as a transmethylator (transfers methyl groups from one molecule to another within a cell) or is involved in major biochemical reactions, such as gene transcription and expression, signaling, cell division and growth, and response to stress. Previous studies have also shown that overexpression of the SAM synthase gene or SAM-related genes in plants or eukaryotes increases tolerance to abiotic stress [14-20]. Therefore, in this study, we constructed and cultivated recombinant *C. reinhardtii* to overexpress the SAMS gene and investigate the cell growth and intracellular lipid accumulation under nitrogen-depleted or saline-stress conditions.

## Materials and Methods

### Strains and Growth Conditions

In this study, *Escherichia coli* DH5α (RBC Bioscience, Taiwan) was used as the gene cloning host, while wild-type *C. reinhardtii* cc-125 was used to evaluate the growth rate, lipid accumulation, and SAM synthase activity. *C. reinhardtii* cells were incubated in Tris-acetate-phosphate (TAP) medium and cultured at 25°C by shaking at 200 rpm under continuous cool white light (40 μmol photons/m^2^/s). The TAP medium contained 2.42 g Tris-base, 25 ml TAP-salts (Beigerinck salts; 15 g/l ammonium chloride [NH_4_Cl], 4 g/l magnesium sulfate heptahydrate [MgSO_4_·7H_2_O], and 2 g/l calcium chloride dehydrate [CaCl_2_·2H_2_O]), 1 ml phosphate solution (288 g/l dipotassium hydrogen phosphate [K_2_HPO_4_] and 144 g/l potassium dihydrogen phosphate [KH_2_PO_4_]), 1 ml of Hutner’s Trace Elements solution (50 g/l disodium ethylenediaminetetraacetic acid dehydrate [Na_2_EDTA·2H_2_O], 22 g/l zinc sulfate heptahydrate [ZnSO_4_·7H_2_O], 11.4 g/l boric acid [H_3_BO_3_], 5 g/l manganese chloride tetrahydrate [MnCl_2_·4H_2_O], 5 g/l ferrous sulfate heptahydrate [FeSO_4_·7H_2_O], 1.6 g/l cobalt chloride hexahydrate [CoCl_2_·H_2_O], 1.6 g/l copper sulfate pentahydrate [CuSO_4_·H_2_O], and 1.1 g/l diammonium molybdate [(NH_4_)_6_MoO_3_]), and 1 ml acetic acid per liter. For the nitrogen-depleted conditions, TAP without nitrogen (TAP-N, in which NH_4_Cl in TAP was replaced with KCl) was used. For the saline-stress conditions, sodium chloride (NaCl) was added to TAP medium at various concentrations (0 (the control), 100, 200, or 300 mM). For either of these two conditions, three-day-old cells were centrifuged at 12,000 ×*g* for 5 min, transferred to either TAP-N or TAP+NaCl medium, and cultured as described above.

### Multiple Sequence Alignment Analysis

The DNA sequence and the amino acid sequence of SAMS for *C. reinhardtii* were obtained from previous report [21]. This amino acid sequence was compared with those of other species (*E. coli* str. K-12 substr. MG1655 (NP_417417.1), *Arabidopsis thaliana* (AAA32868.1), *Saccharomyces cerevisiae* (P10659.2), and *Homo sapiens* (NP_000420.1)) using Cluster Omega software (version 1.2.2).

### Vector Construction and Generation of the Recombinant *C. reinhardtii* Overexpressing SAMS

The DNA sequence for *C. reinhardtii* SAMS was used to fabricate specific primers for cloning (Table 1). These contained the restriction enzyme sites of NcoI and HindIII. DNA fragments corresponding to *C. reinhardtii* SAMS cDNA were amplified via the polymerase chain reaction (PCR) using primers SAMS-F and SAMS-R and a First-Strand cDNA Synthesis Kit (TaKaRa Bio Inc., Japan) according to the manufacturer’s protocol. The plasmid and strains used in this study were described in Table 1. Plasmid pCr102, used as the vector in this study [22], was constructed by inserting *C. reinhardtii* SAMS DNA fragments obtained via digestion with NcoI and HindIII. To transfer the SAMS-overexpression construct into *C. reinhardtii*, cells were incubated in TAP media for 3 days, and when they reached the mid-log phase (3.0 × 10^6^ cells/ml), they were centrifuged at 2,500 ×*g* for 5 min. Cells were resuspended in TAP medium containing 60 mM sucrose. For each cell sample (250 μl), 1 μg of DNA was mixed in a Gene Pulser Cuvette (Bio-Rad, USA), followed by incubation for 5 min at 16°C. Electroporation was executed at 750 V, 25 μF, and a resistance of 200 Ω. Afterward, the cells were incubated for 10 min at room temperature. To recover the cells, they were transferred to TAP with 60 mM sucrose, and then incubated for 1 day with constant shaking at 200 rpm under white light. After recovering them, the cells were plated on TAP agar medium containing hygromycin B (50 μg/ml).

### RNA Isolation

Three-day-old cells were centrifuged at 12,000 ×*g* for 5 min. To isolate the RNA, cells were resuspended with 1 ml Trizol LS Reagent (Ambion Inc., USA), mixed for 10 min by vortexing, incubated at room temperature for 5 min, and centrifuged at 13,000 ×*g* for 10 min at 4°C. The supernatant was mixed with 250 μl of chloroform (Duksan Pure Chemical Co., Korea) by vortexing for 2 min, and centrifuged for 10 min at 4°C. The supernatant was combined with an equal volume of phenol-chloroform (50:50 v/v; Ambion Inc.), mixed for 2 min by vortexing, and then centrifuged. The supernatant was mixed with an equal volume of isopropanol (Daejung Chemical Co., Korea) and incubated for 1 h at 4°C. The mixture was centrifuged for 20 min, after which the precipitated RNA pellet was washed with 70% ethanol (v/v), dried for 5–7 min at room temperature, and then resuspended in diethyl pyrocarbonate water.

### Quantitative Real-Time PCR (qRT-PCR)

To confirm whether the cells were transformed and observe the change of expression, quantitative real-time PCR (qRT-PCR) was carried out. cDNA synthesis was conducted as described above. The primers used in qRT-PCR were described in Table 1. The transcription level was analyzed by using TB Green Premix Ex Taq (TaKaRa) according to the manufacturer’s protocol. Quantification was carried out using a Real-Time PCR system (Illumina Inc., USA) (40 PCR cycles: 10 s at 95°C and 30 s at 58°C). Data were analyzed by using the 2^-ΔΔCt^ method [23]. For quantification, *Tuba1a* was used as the internal control in qRT-PCR.

### Measurement of the Cell Growth and Survival Rates

The *C. reinhardtii* wild-type control (Cr_control) and recombinant *C. reinhardtii* overexpressing SAMS (Cr_SAMS) were cultured in 10 ml TAP broth, after which 4 ml of seed culture was incubated in 200 ml of TAP medium. The other culturing conditions were the same as previously described. The optical density at 750 nm was measured using a UV spectrophotometer (Molecular Devices, USA) every 24 h.

To measure the tolerance of Cr_control and Cr_SAMS to saline stress, 10 μl of cells was washed with 1 × phosphate-buffered saline and then spotted onto TAP agar plates with various concentrations (0, 100, 200, or 300 mM) of NaCl. The cells were cultured at 25°C for 6 days under continuous cool white fluorescent light. The survival rate of *C. reinhardtii* cells was defined as the ratio of colony numbers in the TAP plate containing 0 mM NaCl to those in the TAP plates containing 100, 200, or 300 mM NaCl, respectively.

### SAM Synthetase Activity Assay

For this assay, cells were harvested via centrifugation and disrupted with a bead tube (Zirconia 2mm) lysis kit (MP Bio, USA). The lysates were incubated at 30°C for 3 h in a reaction mixture of 1 M Tris-HCl (pH 8.0), 20 mM MgCl_2_, 1 mM dithiothreitol, 150 mM KCl, 5 mM ATP, and 5 mM L-methionine. The reaction was terminated with 100 mM EDTA [24]. The reaction mixture was analyzed via high-performance liquid chromatography (HPLC; Agilent Technologies Inc., USA) using a ZORBAX SB-C18 column (250 × 4.6 mm, Agilent). The column was eluted with 60% buffer A (water containing 15% methanol and adjusted to pH 3.90 with acetic acid) and 40%buffer B (water). SAM separation was performed in isocratic elution with a flow rate of 0.6 ml/min for 10 min. The mobile phase was degassed to eliminate the dissolved air and injected into the filtration unit by using a 0.22 μm Millipore PTFE membrane [25].

### Microscopic Observations and Lipid Quantification

Nile red (Sigma-Aldrich, USA) was used to stain the cells because lipid droplets were observed under a microscope through fluorescence (Carl Zeiss, Germany). After dissolving the staining reagent in 5% acetone (5 μg/ml), cells were incubated for 30 min at 37°C in the dark [26]. Lipid quantification was measured according to the method [13].

### Extraction of Chlorophyll from *C. reinhardtii*

The extraction of chlorophyll from *C. reinhardtii* was conducted according to the method [27]. The harvested cells were extracted by reaction with 95% ethanol at 78°C for 5 min, after which the supernatant was obtained via centrifugation and measured using a UV spectrophotometer at 656 nm. Relative chlorophyll degradation was defined as the percentage of chlorophyll concentration before nitrogen starvation to that after starvation. Thereafter, the relative chlorophyll degradation concentrations obtained under normal and nitrogen-depleted conditions were compared and expressed as a percentage [13]. GraphPad Prism version 8.00 (GraphPad, USA) was used for statistical analysis, in which Student *t*-tests were performed to test for significant differences in lipid and chlorophyll content. Statistical significance was set as *p* < 0.05.

## Results

### Sequence Alignment Analysis of *C. reinhardtii* SAMS

**Fig. 1:**
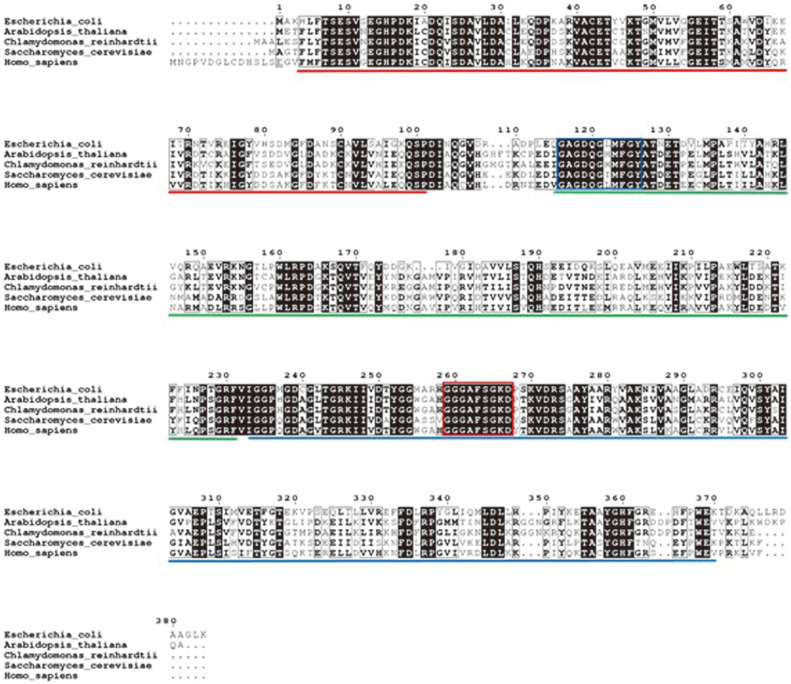
Multiple sequence analysis of SAM synthase from *C. reinhardtii* and other representative species. These were *A. thaliana* (AAA32868.1), *E. coli* str. K-12 substr. MG1655 (NP_417417.1), *S. cerevisiae* (P10659.2), and *H. sapiens* (NP_000420.1). The boxes indicate the binding sites: the blue box for SAM synthase signature motif 1 (active signature for ATP binding site) (PS00376), [GN]-[AS]-G-D-Q-G-x(3)-G-[FYHG] and the red box for SAM synthase signature motif 2 (phosphate-binding region) (PS00377), G-[GA]-G-[ASC]-[FY]-S-x-K-[DE]. The underlined areas indicate the conserved domains: red indicates the SAM synthetase N-terminal domain (PF00438), green indicates the SAM synthetase central domain (PF02772), and blue indicates the SAM synthetase C-terminal domain (PF02773).

The amino acid sequence of SAM synthase of *C. reinhardtii* was compared with those of other species (Fig. 1), namely *E. coli*, *S. cerevisiae*, *H. sapiens*, and *A. thaliana*, in which conservation levels of 56.53, 60.37, 62.40, and 80.88% were obtained, respectively. Fig. 1 shows the conserved regions and substrate binding residues of SAM synthase in *C. reinhardtii* and other species [13, 18]. SAM synthase of *C. reinhardtii* consists of three domains (N-terminal domain, Central domain, C-terminal domain) for S-adenosylmethionine synthesis. It has two SAMS signature motifs, the active signature for the ATP binding site (GAGDQGHMFGY, the consensus sequence for ATP binding) and the phosphate-binding region (GGGAFSGKD, the conserved peptide which forms a P-loop)[18]. From the alignment results, the putative SAMS was shown to possess the necessary motifs for S-adenosylmethionine synthesis and could be functionally expressed in *C. reinhardtii*.

### Construction and Growth Rate of Recombinant *C. reinhardtii* Overexpressing SAMS

**Fig. 2:**
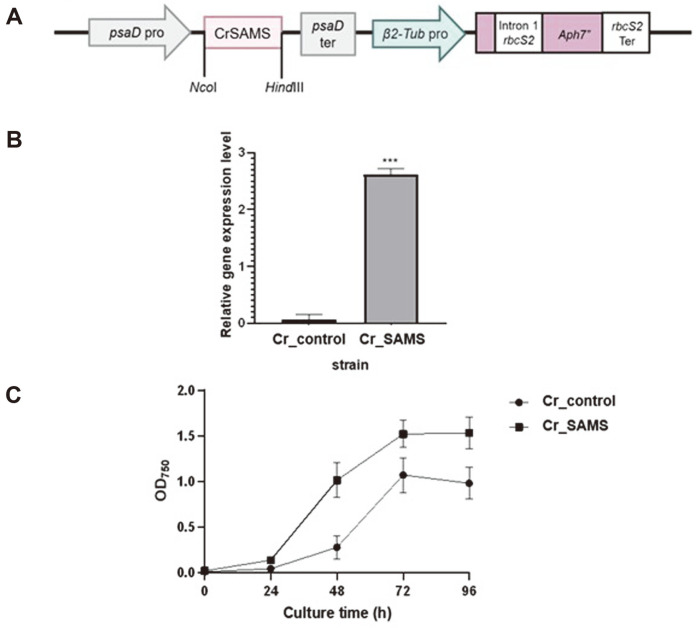
Construction and growth rate of recombinant *C. reinhardtii* overexpressing SAMS. (**A**) A schematic diagram of constructing the SAMS-overexpression vector. (**B**) SAMS transcription levels in recombinant and wild-type *C. reinhardtii*. (**C**) Optical density at 750 nm according to culturing time of the wild-type (Cr_control) and Cr_SAMS cells in TAP medium. Data are expressed as the mean ± standard deviation; *n* = 3; and statistical analysis was carried out using Student *t*-tests: ***p < 0.001.

To overexpress SAMS and investigate the effect of the overexpression, we constructed the recombinant plasmid pCr102-CrSAMS (Table 1), which contained the *psaD* promoter and terminator for SAMS expression, the β-tubulin promoter for *Aph7"* expression, and the *rbcs2* terminator (Fig. 2A). Subsequently, wild-type *C. reinhardtii* cc-125 was used for electroporation, while qRT-PCR was used to select recombinant strains in which pCr102-CrSAMS had been overexpressed (Fig. 2B). From the results of comparing the gene expression between Cr_SAMS and Cr_control, it was confirmed that recombinant *C. reinhardtii* Cr_SAMS showed a 3-fold higher expression of SAMS. To confirm the overexpression of SAMS, Cr_SAMS and Cr_control were cultivated in the presence or absence of nitrogen for 3 or 7 days, and then the activity of SAMS was analyzed by HPLC. After culturing in TAP medium for 3 or 7 days, neither Cr_SAMS nor Cr_control showed significantly different SAMS activity. However, after culturing in nitrogen-limited TAP media for 3 or 7 days, a 10% increase in activity of SAMS was observed in Cr_SAMS compared to Cr_control (data not shown).

The growth rates of Cr_SAMS and Cr_control were compared to determine whether these processes were enhanced by overexpressing the SAMS gene in *C. reinhardtii*. The cell density was observed each day while culturing Cr_SAMS and Cr_control in TAP medium for 4 days. In Fig. 2C, a difference between the cell densities of the two strains was revealed after incubation for 2 days. When the final cell densities were compared after cultivation for 4 days, the OD_750_ values of 1.53 for Cr_SAMS and 0.98 for Cr_control indicated a growth increase of 1.56-fold in Cr_SAMS.

### Comparison of Chlorophyll Degradation and Lipid Accumulation in Nitrogen-Depleted Conditions

**Fig. 3:**
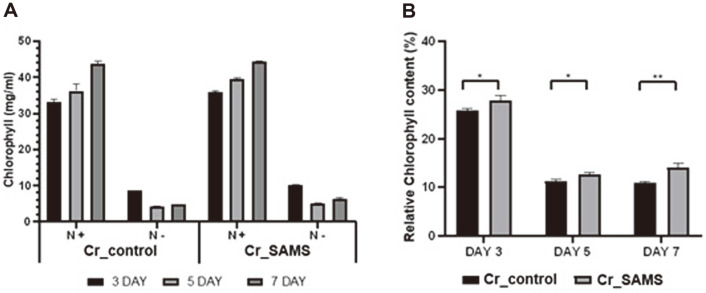
Quantitative analyses of chlorophyll contents of Cr_SAMS and Cr_control. (**A**) Chlorophyll content under normal and nitrogen-depleted conditions and (**B**) the relative chlorophyll degradation under nitrogen-depleted conditions. Data are expressed as the mean ± SD; *n* = 3; statistical analysis was carried out using Student’s t-tests: *p < 0.05, **p < 0.005.

To evaluate the effect of SAMS overexpression by *C. reinhardtii* under nitrogen limitation conditions, the amounts of chlorophyll in Cr_SAMS and Cr_control were observed. The results were presented in Fig. 3A. When cultured in TAP medium, both Cr_SAMS and Cr_control showed increasing amounts of chlorophyll as the cultivation period was increased, with that in the former being slightly higher. When cultured in nitrogen-depleted medium, both strains showed a sharp decrease in the concentration of chlorophyll after 5 days of cultivation compared to after 3 days. Under nitrogen-depleted conditions, *C. reinhardtii* is characterized by chlorophyll degradation [9]. When comparing the degree of chlorophyll degradation between the two strains, that in Cr_SAMS was less than that in Cr_control (Fig. 3B). The relative chlorophyll concentrations of Cr_SAMS and Cr_control were 27.9% and 25.8% after culturing for 3 days under nitrogen-limited conditions, respectively. Furthermore, the relative chlorophyll concentrations were significantly decreased to 12.6% and 11.3% in Cr_SAMS and Cr_control after culturing for 5 days, respectively. When the cells were cultivated for 7 days under nitrogen-limited conditions, the relative chlorophyll concentrations were measured to be 14.1% and 10.9% in Cr_SAMS and Cr_control, respectively. These results suggest that the overexpression of SAMS could cause less degradation of chlorophyll and increase the production of metabolites in the cells.

**Fig. 4:**
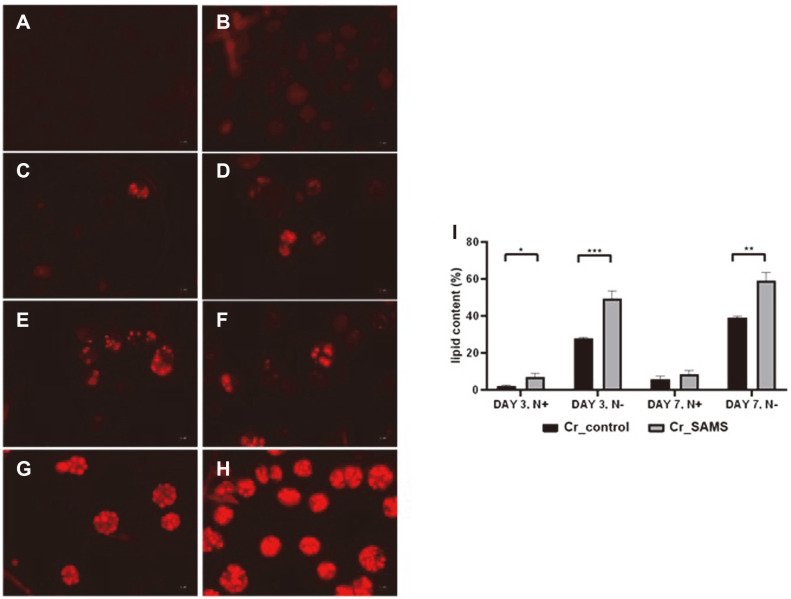
Nile red-stained microscopic images and quantitative analysis of lipids in Cr_SAMS and Cr_control under normal and nitrogen-depleted conditions. (**A, B**) Under normal conditions for 3 days, (**C, D**) under normal conditions for 7 days, (**E, F**) under nitrogen-depleted conditions for 3 days, and (**G, H**) under nitrogen-depleted conditions for 7 days, respectively. (**I**) The quantitative data for lipid content. Data are expressed as the mean ± SD; *n* = 3; statistical analysis was carried out using Student’s t-tests: *p < 0.05, **p < 0.005, ***p < 0.001. Scale bar = 1 μm.

Lipid production in Cr_SAMS and Cr_control was confirmed qualitatively and quantitatively. Since *C. reinhardtii* accumulates lipids in the body under nitrogen-depleted conditions, lipid accumulation was observed after culturing Cr_SAMS and Cr_control for 3 and 7 days under normal and nitrogen-depleted conditions (Fig. 4). When cultured for 3 days, Cr_SAMS and Cr_control showed lipid contents of 7.16% and 2.26% in normal TAP medium, respectively. However, after cultivation for 7 days, the lipid content of Cr_SAMS and Cr_control was 8.57% and 5.82% in the normal medium, respectively. The production rate of lipids was observed to be much faster in *C. reinhardtii* overexpressing SAMS without nitrogen limitation. Meanwhile, the effect of SAMS overexpression was more significant under nitrogen limitation conditions. When Cr_SAMS and Cr_control were cultivated in a nitrogen-depleted medium, the lipid content was 49.6% and 28.1% for 3 days, respectively. After cultivation for 7 days, the lipid content of Cr_SAMS and Cr_control was 59.2% and 39.3% in the nitrogen-depleted medium, respectively. Thus, Cr_SAMS showed 1.51 times higher lipid production compared to control in the nitrogen-depleted medium. However, the difference between the two strains was not significant when comparing cell growth under nitrogen-depleted conditions.

### Comparison of the Cell Survival Rate and Lipid Accumulation under Saline-Stress Conditions

**Fig. 5:**
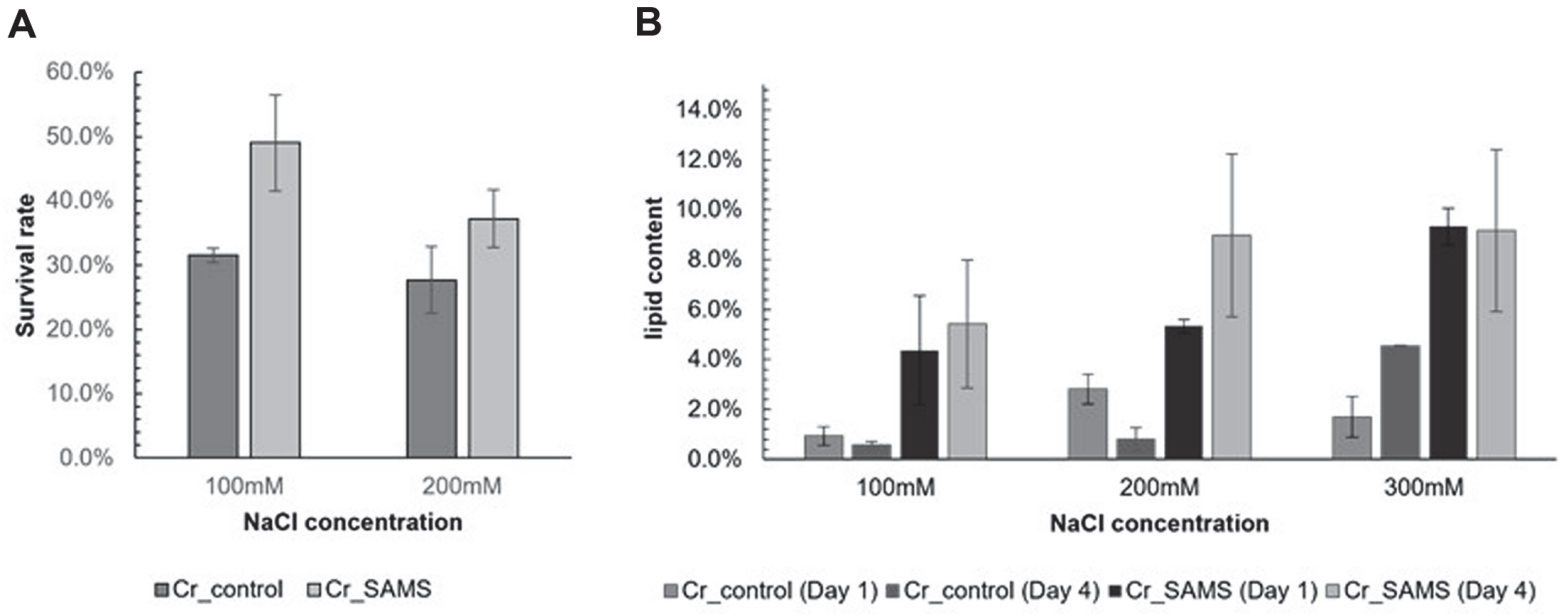
Quantitative analyses of Cr_SAS1 and Cr_control cells under NaCl-stress conditions. (**A**) Survival rates in TAP media containing 100 mM or 200 mM NaCl. (**B**) Lipid content in TAP media containing various concentrations of NaCl. Data are expressed as the mean ± SD; *n* = 3.

To investigate the possible application of recombinant *C. reinhardtii* overexpressing SAMS for production of lipids in salt water, cell survival rate and lipid production were measured under saline-stress conditions. After spotting Cr_SAMS and Cr_control on a TAP agar plate supplemented with 0, 100, 200, or 300 mM NaCl, respectively, the survival rates were determined by comparing the number of colonies (Fig. 5A). At a concentration of 300 mM NaCl, no colonies were formed in Cr_SAMS or Cr_control. At 100 mM and 200 mM salinity, the survival rates for Cr_SAMS were 49.0% and 37.2%, respectively, while those for Cr_control were 31.5% and 27.7%, respectively. The differences in survival rate were 1.56 times and 1.34 times, respectively, showing that the survivability was significantly increased by overexpressing SAMS under saline-stress conditions.

The amount of lipid accumulation under saline-stress conditions was also measured and compared between strains. After the growth phase, Cr_SAMS and Cr_control were cultured under saline-stress conditions. On day 1, Cr_SAS1 and Cr_control showed lipid levels of 4.4% and 0.9% in 100 mM NaCl-supplemented TAP medium. The concentrations were increased to 5.3% and 2.8%, respectively, in 200 mM NaCl-supplemented TAP medium. When cultivated in 300 mM NaCl-supplemented TAP medium, Cr_SAMS and Cr_control accumulated lipid at levels of 9.3% and 1.7%, respectively (Fig. 5B). After cultivation for 4 days, Cr_SAMS and Cr_control showed lipid content of 5.4% and 0.6% in 100 mM NaCl-supplemented TAP medium, 9.0% and 0.8% in 200 mM NaCl-supplemented TAP medium, and 9.2% and 4.5% in 300 mM NaCl-supplemented TAP medium, respectively (Fig. 5B).

## Discussion

SAM is a substance that acts as an important molecule in organisms in which methionine and ATP are biosynthesized through SAMS [18]. In this study, through sequence alignment, we confirmed that SAMS of *C. reinhardtii* has conserved residues shared by SAMS of other prokaryotes and eukaryotes, and overexpression was carried out in *C. reinhardtii* (Fig. 1). The expression level of SAMS was significantly increased in the recombinant, though no significant difference in activity between the wild-type and recombinant mutant *C. reinhardtii* was observed. Turning mRNA into protein sometimes has no correlation, because there are many complicated translation and post-translational mechanisms, and proteins are different in their in vivo half-life [28]. The recombinant strain could have a stability problem since the transient expression is an issue for gene expression in *Chlamydomonas*. In this study, however, the expression levels of SAMS were checked, and there was no significant difference after cultivation.

SAM is used as a precursor for polyamine compounds. A carboxyl group is removed from SAM by S-adenosylmethionine decarboxylase (SAMDC), and then decarboxylated SAM (dcSAM) is used in the pathway for synthesizing polyamine compounds. Polyamines play essential functions in cell growth and division, such as protecting nucleic acids, regulating gene expression, protein translation and signal transduction, and stabilizing cell membranes in microalgae [29]. This suggests that the increase in SAM production through overexpression of SAMS activates the polyamine metabolic pathway, affecting both cell division and cell growth [14, 30, 31] (Fig. 2C).

Polyamines have been confirmed to play an important role in abiotic stress. Abiotic stress causes damage to various cellular components, such as cell membranes, proteins, and lipids by generating reactive oxygen species (ROS) in microalgae and plants [32, 33]. Polyamines have shown stress tolerance by regulating the antioxidant system or inhibiting ROS generation [34]. In previous studies, the resistance to abiotic stress increased when plants actually overexpressed genes related to polyamine metabolism pathways such as SAMDC [19, 20, 29]. Therefore, through the overexpression of SAMS, we confirmed that the microalga *C. reinhardtii* also increased the stress tolerance of cells through the function of polyamine (Fig. 5B). In Fig. 5, overexpression of SAMS in *C. reinhardtii* increased cell resistance to saline stress among abiotic stresses, thereby confirming a positive effect in terms of lipid production. This can be an advantage in producing biofuel through the cultivation of microalgae in saline water. In addition, polyamines synthesized from microalgae are related to chlorophyll proteins, and play an important role in photosynthesis [29, 31]. As demonstrated in Fig. 3, the overexpression of SAMS reduces relative chlorophyll degradation under nitrogen depletion conditions. The carbon source for lipid accumulation in microalgae is derived from CO_2_ fixed through photosynthesis. Thus, under nitrogen-depleted conditions, the relatively less degraded chlorophyll fixes a higher amount of carbon, explaining the difference in lipid accumulation [4, 35, 36] (Fig. 4). This result can also be an advantage when using a two-phase cultivation strategy in terms of biofuel production with microalgae [6]. Therefore, we confirmed that overexpressing SAMS in the strain *C. reinhardtii* is advantageous in terms of cell growth and lipid production under nitrogen depletion stress and salinity stress.

In this study, overexpressing SAMS in *C. reinhardtii* increased cell growth 1.56-fold compared to the wild-type strain. Moreover, lipid accumulation was increased to 1.76-fold in nitrogen-limited medium. In addition, under nitrogen-depleted conditions, the relative chlorophyll content was 1.5-fold higher in the SAMS-overexpressed strain. Under saline-stress conditions, the survival rate and lipid accumulation were 1.56 times and 2.04 times higher in the SAMS-overexpressing strain, respectively. The results of this study suggest that overexpression of SAMS in *C. reinhardtii* could enhance the cell growth and lipid accumulation under abiotic conditions. Further, they indicate the high potential of *C. reinhardtii* for industrial-scale production of not only biofuel but also other useful substances.

## Figures and Tables

**Table 1:** Plasmids, strains and primers used in this study.

Plasmid, strains	Characteristics		Source

Plasmid			
pCr102	*psaD* promoter and terminator, β-tubulin promoter for *Aph7"* expression, and *rbcs2* terminator		[22]
pCr102-CrSAMS			This study
Strain			
Cr_control	*C. reinhardtii* Wild type (cc-124)		[37]
Cr_SAMS	Cr_control/pCr102-CrSAMS		This study
*E. coli* DH5α	Gene-cloning host		RBC

Primers for cDNA cloning			

Primer	Primer Sequence	Target Gene	

SAMS-F	ATCCCATGGATGGCCGCCCTGGAGTCCT	SAS1 cDNA amplification	
SAMS-R	ATCAAGCTTTTACTCCAGCTTCTTCAC		

Primers for qRT-PCR			

Primer	Primer Sequence	Target Gene	

tubA-F	CTC GCT TCG CTT TGA CGG TG	*Tuba1a* *	
tubA-R	CGT GGT ACG CCT TCT CGG C		
qSAMS-F	ATCCCATGGATGGCCGCCCTGGAGTCCT	SAS1	
qSAMS-R	ATCAAGCTTTTACTCCAGCTTCTTCAC		

*The internal control
